# Supplementary figures and images for: The evolution of unique cranial traits in leporid lagomorphs
Source: PeerJ. 2022 Nov 29;10:e14414. doi: 10.7717/peerj.14414 (PMC9744148; doi:10.7717/peerj.14414)

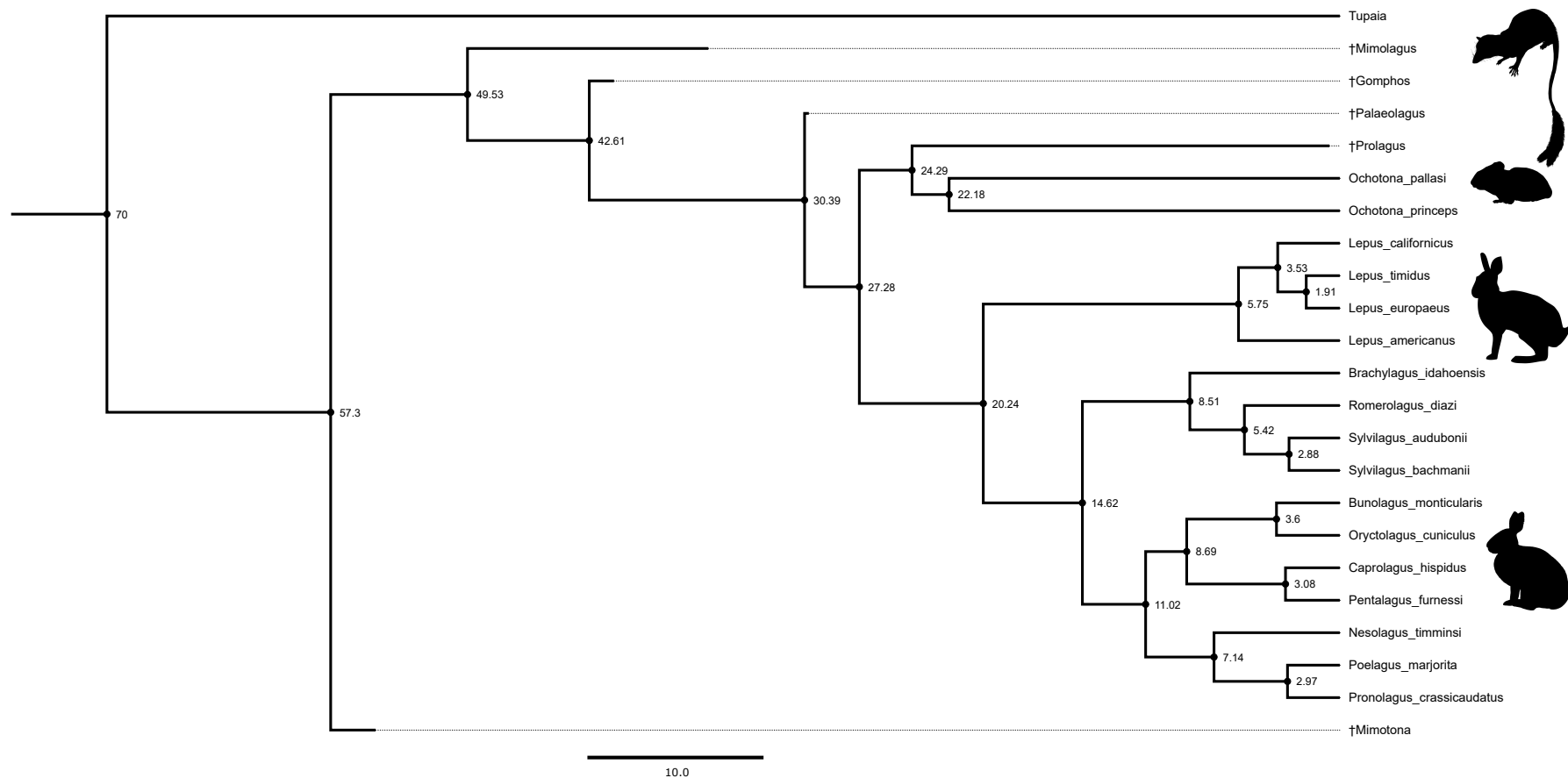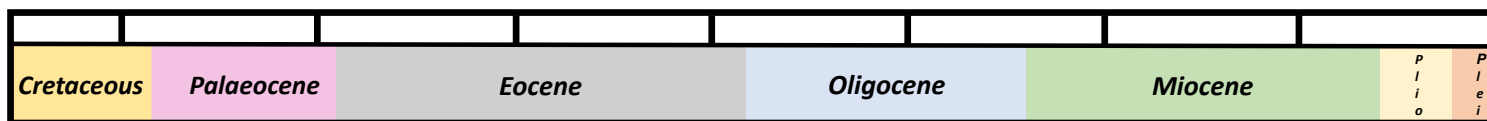

Supplement: Supplemental Information 4 — The tree includes at least one member of every extant genus of lagomorph. Fossil taxa are denoted with “†”. The values at internal nodes are the median divergence time estimates at key nodes. Tupaia and Ochotona silhouettes used under Public Domain Dedication 1.0 license. Sylvilagus and Lepus silhouettes used under Creative Commons Attribution-NonCommercial 3.0 Unported license with credit given to Gabriela Palomo-Munoz and Sarah Werning. Image credits: Phylopic: T. Michael Keesey (after Joseph Wolf), Public Domain Dedication 1.0 (https://creativecommons.org/publicdomain/zero/1.0/): http://phylopic.org/image/88a07585-846a-405d-9195-c15c010e7443/; Margot Michaud, Public Domain Dedication 1.0 (https://creativecommons.org/publicdomain/zero/1.0/): http://phylopic.org/name/79021e04-3b8d-4a49-9902-24d744d4af51; Gabriela Palomo-Munoz, Creative Commons Attribution-NonCommercial 3.0 Unported (https://creativecommons.org/licenses/by-nc/3.0/): http://phylopic.org/image/24535087-a458-4d5e-ad59-eba10a4cd080/; Sarah Werning, Creative Commons Attribution 3.0 Unported (https://creativecommons.org/licenses/by/3.0/), http://phylopic.org/image/dea688b6-9168-4e79-a106-366888148eb1/. [file peerj-10-14414-s004.pdf]
